# Supplementary material for: Content-rich biological network constructed by mining PubMed abstracts
Source: BMC Bioinformatics. 2004 Oct 8;5:147. doi: 10.1186/1471-2105-5-147 (PMC528731; doi:10.1186/1471-2105-5-147)
Supplement: Additional File 2 — The original results of the above study (non-essential files are deleted to keep the file size under the limit set by BMC bioinformatics). [file 1471-2105-5-147-S2.bz2 › chilibotAdditionalFile2/dip05/387ID11297552E9861/html/PDGFA_PDGFRA.html]

 


 **PDGFA** and **PDGFRA** 
  
Found 8 abstracts in PubMed, retrieved 05.  
 

 What does Google say? 
 PDF only 
| .edu only 

---

- Przegl Lek, 2002    **Structure and function of PDGF R alpha **[PDGFRA]** and its expression in normal kidney and kidney diseases** .
  Platelet derived growth factor is commonly known as a mitogen.
  Many research data suggest a role for PDGF beta R in the mitogenic response of mesangial cells.
  There are four members of PDGF family known as PDGF A chain **[PDGFA]**, PDGF B chain, PDGF C chain and PDGF D chain, which in active forms are dimers.
  As far as two receptors PDGF alpha R and PDGF beta R are known to bind PDGF.
  There is a difference in binding affinity of various forms of PDGF by these receptors.
  Two different promotors P1 and P2 can be used for PDGF alpha R gene transcription.
  There are several different haplotypes of promotor P1 sequence.
  Transcription of PDGF alpha R gene is under control of many factors.
  Interaction between a receptor and its ligand includes receptor dimerisation and autophosphorylation of tyrosine residues.
  PDGF AA is unique in that it can only be bound by alpha receptor dimer.
  PDGF AA expression has been confirmed in the normal kidney, as well as in several renal diseases.
  Although the expression of PDGF alpha R has been found to accompany that of PDGF AA, its actual relevance for the development of the glomerular pathology is not clear.

  - Development, 2002   **Dual origin of spinal oligodendrocyte progenitors and evidence for the cooperative role of Olig2 and Nkx2.2 in the control of oligodendrocyte differentiation.** .
    In this study, we have investigated the relationship of Olig2 and Nkx2.2 oligodendrocyte progenitors OLPs by comparing the expression of Olig2 and Nkx2.2 in embryonic chicken and mouse spinal cords before and during the stages of oligodendrogenesis.
    At the stages of neurogenesis, Olig2 and Nkx2.2 are expressed in adjacent non overlapping domains of ventral neuroepithelium.
    During oligodendrogenesis stages, these two domains generate distinct populations of OLPs.
    From the Olig2 motoneuron precursor domain pMN arise the Olig2 **Pdgfra** OLPs.
    . . . the Nkx2.2 p3 domain give rise to Nkx2.2 OLPs.
    Despite their distinct origins, both populations of OLPs eventually appear to co express Olig2 and Nkx2.2 in the same cells.
    However, there is a species difference in the timing of acquiring Nkx2.2 expression by the Olig2 **Pdgfra** OLPs.
    The co expression of Nkx2.2 and Olig2 in OLPs is tightly associated with myelin gene expression in the normal and **PDGFA** embryos, suggesting a cooperative role of these transcription factors in the control of oligodendrocyte differentiation.
    In support of this suggestion, inhibition of expression of these two transcription factors in culture by antisense oligonucleotides has an additive inhibitory effect on OLP differentiation and proteolipid protein PLP gene expression.

    - Nat Genet, 2001   **Expression profiling of medulloblastoma **PDGFRA** and the RAS MAPK pathway as therapeutic targets for metastatic disease.** .
      Little is known about the genetic regulation of medulloblastoma dissemination, but metastatic medulloblastoma is highly associated with poor outcome.
      We obtained expression profiles of 23 primary medulloblastomas clinically designated as either metastatic M or non metastatic M0 and identified 85 genes whose expression differed significantly between classes.
      Using a class prediction algorithm based on these genes and a leave one out approach, we assigned sample class to these tumors M or M0 with 72% accuracy and to four additional independent tumors with 100% accuracy.
      We also assigned the metastatic medulloblastoma cell line Daoy to the metastatic class.
      Notably, platelet derived growth factor receptor alpha **[PDGFRA]** **PDGFRA** and members of the downstream RAS mitogen activated protein kinase MAPK signal transduction pathway are upregulated in M tumors.
      Immunohistochemical validation on an independent set of tumors shows significant overexpression of **PDGFRA** in M tumors compared to M0 tumors.
      Using in vitro assays, we show that platelet derived growth factor alpha **[PDGFA]** **PDGFA** enhances medulloblastoma migration and increases downstream MAP2K1 MEK1 , MAP2K2 MEK2 , MAPK1 p42 MAPK and MAPK3 p44 MAPK phosphorylation in a dose dependent manner.
      Neutralizing antibodies to **PDGFRA** blocks MAP2K1, MAP2K2 and MAPK1 3 phosphorylation.
      . . . U0126, a highly specific inhibitor of MAP2K1 and MAP2K2, also blocks MAPK1 3.
      Both inhibit migration and prevent **PDGFA** stimulated migration.
      These results provide the first insight into the genetic regulation of medulloblastoma metastasis and are the first to suggest a role for **PDGFRA** and the RAS MAPK signaling pathway in medulloblastoma metastasis.
      Inhibitors of **PDGFRA** and RAS proteins should therefore be considered for investigation as possible novel therapeutic strategies against medulloblastoma.

      - Mol Hum Reprod, 2001   **Protein tyrosine kinase expression in the porcine ovary.** .
        Various growth factor receptors contain intrinsic tyrosine kinase activity, indicating that protein tyrosine kinases PTK play an important role in signal transduction pathways for cell proliferation and differentiation.
        To identify oocyte derived factors which control follicle cells as well as oocyte controlling factors produced by follicle cells, we examined the expression of genes which contain the PTK domain in the porcine ovary, using a polymerase chain reaction based amplification technique with degenerate oligonucleotide primers that are specific to the PTK domain.
        Clones for the porcine homologues of platelet derived growth factor receptor alpha **[PDGFRA]** PDGFRalpha and of insulin like growth factor I receptor IGF IR were found during follicle growth both in oocytes and follicle cells.
        Clones for the porcine homologues of focal adhesion kinase FAK , of c kit and of fms like tyrosine kinase FLT 3 were found only in oocytes.
        Moreover, after 24 h of in vitro maturation of the cumulus oocyte complexes, clones for the porcine homologues of FLT 1, of FLT 4, of Tie2 and of RYK in oocytes were observed.
        Immunohistochemical studies revealed the existence of PDGFRalpha, platelet derived growth factor A **PDGFA** , FAK and FLT3 in oocytes at various stages of folliculogenesis.
        These results suggest that fluctuations in the expression of these PTK genes may be involved in follicle growth and maturation.

        - Arthritis Rheum, 2000   **Microsatellites and intragenic polymorphisms of transforming growth factor beta and platelet derived growth factor and their receptor genes in Native Americans with systemic sclerosis scleroderma a preliminary analysis showing no genetic association.** .
          OBJECTIVE Abnormalities of transforming growth factor beta TGFbeta and platelet derived growth factor PDGF alpha and beta and or their receptors have been demonstrated in systemic sclerosis SSc .
          This study aimed to determine whether genetic polymorphisms in or near the TGFbeta and PDGF gene families were associated with susceptibility to SSc in a Native American population with a high disease prevalence.
          METHODS Genotyping of 5 intragenic polymorphisms within the TGFbeta1 gene and mapping of 35 microsatellites near the genes for TGFbeta1, latent TGFbeta1 binding protein LTBP1 , TGFbeta receptors I and II, PDGFalpha, PDGFbeta, PDGF receptor alpha, and PDGF receptor beta was performed in 19 SSc patients, 76 controls, and 42 family members.
          Allele distributions and frequencies were examined between SSc patients and controls, and marker haplotypes were examined in families when allele frequencies appeared to be different between patients and controls.
          RESULTS Although 1 polymorphism within the TGFbeta1 gene TGFbeta1 was modestly increased in the SSc patients, this did not maintain statistical significance after correction.
          Similarly, 1 microsatellite D9S120 near the TGFbeta receptor I gene TGFBR1 showed a significant disturbance of allele frequencies between patients and controls.
          it did not form a disease associated haplotype with other nearby markers.
          Weak disturbances of markers near PDGFalpha **PDGFA** and PDGFbeta, PDGFB also failed to maintain significance after correction.
          Both PDGF receptor genes **PDGFRA** and PDGFRB also showed no disease associations.
          CONCLUSION The results of these preliminary analyses suggest that genetic anomalies of the TGFbeta1 and PDGF gene families are not likely to explain the dysregulation seen in SSc or to account for the susceptibility to SSc in this population.
